# Analysis of Approaches in the Microsurgical Treatment of 102 Cases of Petroclival Meningioma in a Single Center

**DOI:** 10.3389/fneur.2021.627736

**Published:** 2021-03-19

**Authors:** Yazhou Lin, Qiang Gao, Huiping Jin, Nana Wang, Dingkang Xu, Fang Wang, A. Bao Guo, Weidong Zang, Zhihua Li, Fuyou Guo

**Affiliations:** ^1^College of Basic Medical Sciences, Zhengzhou University, Zhengzhou, China; ^2^Department of Neurosurgery, First Affiliated Hospital of Zhengzhou University, Zhengzhou, China

**Keywords:** petroclival, meningioma, skull base, surgical approach, tumor subtypes

## Abstract

**Objectives:** We identified the optimal approaches for treating the diverse tumor subtypes of petroclival meningioma (PM) by analyzing the clinical benefits of various surgical approaches adopted for each subtype.

**Methods:** Tumors in 102 PM patients from a single center who underwent surgical treatment were classified as upper clivus (UC), cavernous sinus (CS), tentorium (TE), or petrous apex (PA) types based on the attachment site of the tumor base and the displacement of the trigeminal nerve. The therapeutic effects of different surgical approaches among the subtypes were evaluated according to the patient outcomes.

**Results:** The subtemporal (33.33%), retrosigmoid (16.67%), and Kawase approaches (50%) were used for the UC type. Simpson I/II resection was achieved in 46.66% of patients with the Kawase approach. Significant differences were found between the other two approaches (*P* = 0.044) and in the follow-up Karnofsky performance scale (KPS) scores (*P* = 0.008). The subtemporal (60%) and Kawase approaches (40%) were used for the CS type; neither approach achieved Simpson I/II resection. The retrosigmoid (25.81%) and Kawase approaches (74.19%) were used for the TE type. The Simpson I/II resection rates of the two approaches were 55.55 and 86.95%, respectively, and a significant difference was observed between them (*P* = 0.039). The retrosigmoid (43.75%) and Kawase approaches (56.25%) were used for the PA type. The Simpson I/II resection rates of the two approaches were 31.25 and 50%, respectively. The resection degrees of the two approaches and the KPS scores at follow-up were significantly different (*P* = 0.034).

**Conclusion:** The individual microsurgical approaches adopted for the various PM tumor subtypes can provide maximal safe resection and good KPS scores. The Kawase approach is more suitable for PM, especially for UC- and PA-type PM tumors.

## Introduction

Petroclival meningioma (PM) is a rare benign tumor that occurs on the skull base. It originates at the upper two-thirds of the clivus medial to the fifth cranial nerve (CN V) and accounts for 2% of posterior fossa meningiomas (1–4). Although it is a benign tumor, due to its deep location and proximity to the brain stem, cavernous sinus (CS), basilar artery and other important structures, upward invasion of the petrous apex (PA), cerebellar notch, Meckel cave, parasellar region, and CS can occur, downward and lateral invasion can involve the inner auditory canal and even the jugular foramen, and growth toward the midline can involve the brainstem, basilar artery, and III-XI cranial nerves on one side (5–8). Therefore, PM is one of the most challenging diseases in neurosurgery due to its high risk and complications. In previous reports, the incidence of permanent cranial nerve injury has ranged from 20.3 to 76%, and the proportion of total tumor resection has varied from 20 to 85% (9–11). Therefore, it has become the common goal of all neurosurgeons to select the appropriate surgical approach according to the invasive site of the tumor base and to reduce postoperative neurological impairments in the patient and improve his/her quality of life while removing as much of the tumor as possible.

Here, we reviewed the surgical treatment of PM patients in combination with magnetic resonance imaging (MRI) findings and microscopic surgery performed to identify tumor base areas. We also analyzed the different surgical approaches and evaluated the tumor growth area and its relationship with the surgical approach.

## Materials and Methods

This retrospective study was approved by the medical ethics committees of institutions I and II and were conducted in accordance with the relevant guidelines. All patient data were collected through the electronic medical record system of the First Affiliated Hospital of Zhengzhou University. Informed consent was waived.

### Patient Population

In total, 102 cases of benign (WHO I grade) PM were confirmed by surgery and pathology in the Department of Neurosurgery of the First Affiliated Hospital of Zhengzhou University. All procedures were approved by the Ethics Committee for Human Experiments of Zhengzhou University. The patients included 21 (20.6%) males and 81 (79.4%) females, with a mean age of 52.3 ± 9.9 years (Table 1). None of the patients had a history of radiation therapy, and 2 patients underwent petroclival meningioma resection in another hospital.

**Table 1:** Summary of general characteristics in patients with PM.

**Characteristics**		
**Age, years**		
	Mean ± SD	52.3 ± 9.9
	Range	26–74
**Sex**, ***n*** **(%)**		
	Male	21 (20.6)
	Female	81 (79.4)
**Duration of disease, months**		
	Mean ± SD	17.0 ± 27.6
	Range	0.3–156
**CN invasion**, ***n*** **(%)**		
	CN II	3 (2.9)
	CN III	4 (3.9)
	CN IV	5 (4.9)
	CN V	18 (17.7)
	CN VI	6 (5.9)
	CN VII	7 (6.9)
	CN VIII	10 (9.8)
	CN IX	6 (5.9)
	CN X	4 (3.9)
	CN XI	4 (3.9)
	None	35 (34.3)
**Symptom**, ***n*** **(%)**		
	Headache or dizziness	55 (53.9)
	Epilepsy	1 (1)
	Dysarthria	11 (10.8)
	Medical examination	35 (34.3)
Recurrence, *n* (%)		8(7.8)
Follow-up, *n* (%)		97 (96)
**Type of tumor**, ***n*** **(%)**		
UC		30 (29.4)
CS		25 (24.5)
TE		31 (30.4)
PA		16 (15.7)
**Mean diameter** ± **SD, mm**		
UC		29.1 ± 6.47
CS		35.8 ± 6.49
TE		27.6 ± 11.32
PA		26.3 ± 12.8

*CS, cavernous sinus type; CN, Cranial nerve; PA, petrous apex type; PM, Petroclival meningioma; TE, tentorium type; UC, upper clivus type*.

All patients were examined by CT or MRI. The formula for calculating the tumor equivalent diameter (TED) is (D1 × D2 × D3)^1/3^, with results categorized as small (<10 mm), medium (10–24 mm), large (25–44 mm) and massive (≥45 mm) according to the diameter (12).

### Classification of Tumors

**Figure 1:**
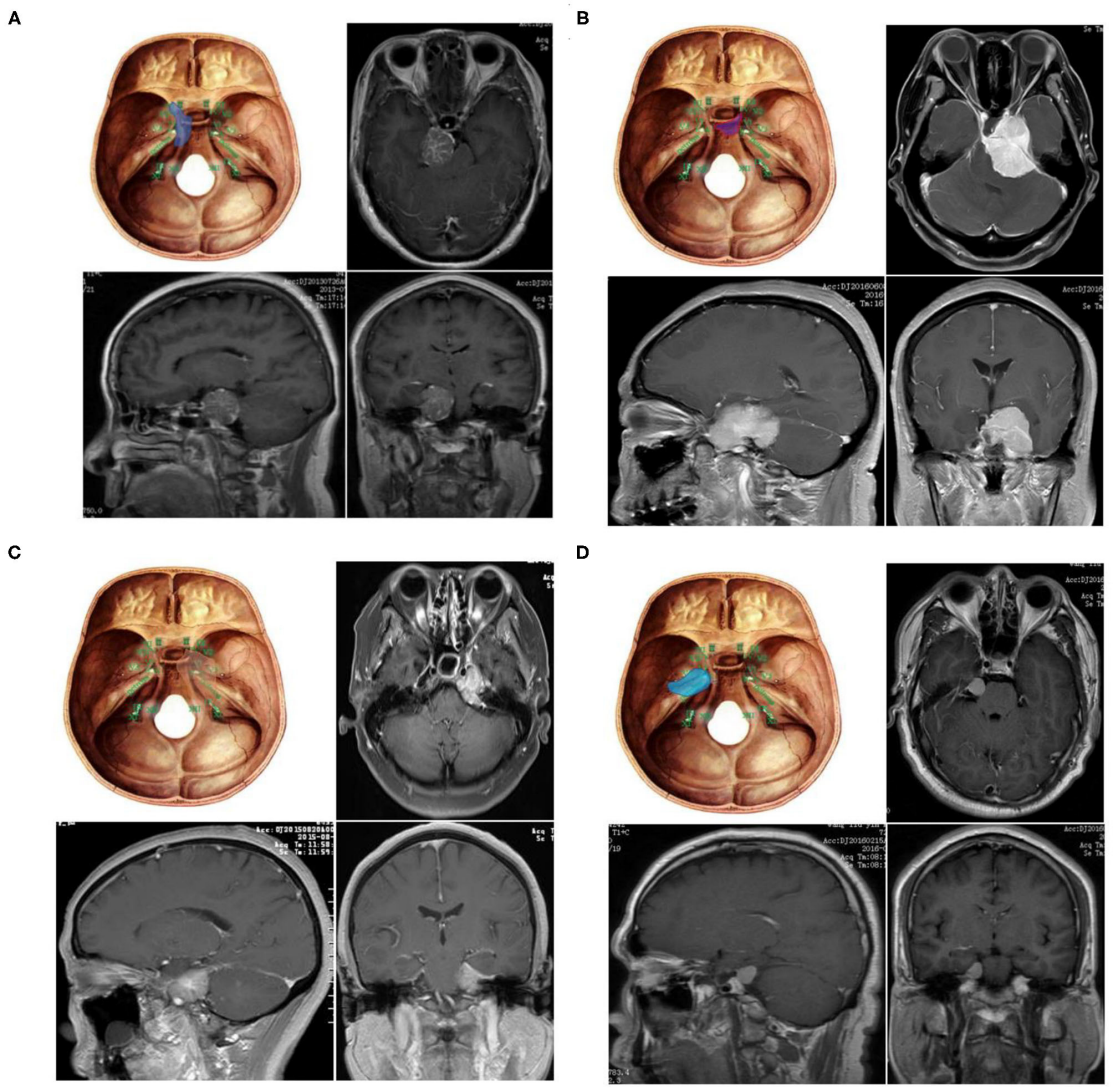
Four subtypes of petroclival meningioma **(A)** Upper clivus type petroclival meningiomas (UC). **(B)** Cavernous sinus type petroclival meningiomas (CS). **(C)** Tentorium-type petroclival meningioma (TE). **(D)** Petrous apex type petroclival meningiomas (PA).

According to the tumor origin combined with the clinical symptoms and the relationship between the petroclival tumor and the trigeminal nerve as evidenced by the Kawase approach (13), PM is divided into four types: 1, upper clivus type (UC): originating from the medial side of the clival trigeminal nerve; 2, cavernous sinus type (CS): originating from the clivus, with subbell-shaped extension to the cavernous sinus; 3, tentorial type (TE): originating from the tentorial trigeminal nerve; and 4, petrous apex type (PA): petrous apex originating from the lateral trigeminal nerve (Figure 1).

### Choice of Surgical Approach and Perioperative Management

This group mainly adopts the subtemporal approach, retrosigmoid approach and Kawase approach.

The subtemporal approach, also called the subtemporal transtentorial approach, uses a horseshoe-shaped incision in the temporal occipital region, creating the lowest edge of the bone flap as low as possible. After successful craniotomy, the skull is entered along the cerebellar tentorium behind the petrous crest. The free margin of the tentorial cerebellum is located, the cistern is opened, and the trochlear nerve is identified and protected. The tumor is then located along the free edge. Small supratentorial tumors are removed first after protecting the oculomotor nerve. For larger tumors, the trochlear and trigeminal nerves are identified and protected as soon as possible.

In the retrosigmoid approach, the lateral prone position is used. The mastoid and asterion plane were used as the highest point of the incision, and the asterion was used as the upper 3/4 point of the straight incision. The transverse sinus, sigmoid sinus and their intersection are fully exposed during craniotomy, and injury to the iatrogenic venous sinus is avoided as much as possible. After opening the dura mater, the cerebellomedullary cistern is dissected, fully releasing cerebrospinal fluid to expose the tumor. In the process of tumor resection, attention should be paid to tumors pushing the VII-VIII cranial nerves backward and inferior. Tumors invading Meckel's cave, the cavernous sinus and middle cranial fossa can be treated by first grinding the medial petrous apex and suprameatal bone.

For the improved Kawase approach developed by Day JD (14), a frontotemporal arc incision is made, the dura of the temporal lobe is elevated, and the bone at the Kawase triangle is milled. The maximum grinding area of the petrous apex area extends to the rhomboid area formed by the trigeminal root foramen, the intersection of the arcuate eminence and the petrosal crest, the geniculate ganglion, the superficial petrosal nerve and the mandibular nerve. The trigeminal ganglion and mandibular nerve are exposed on the anteromedial side, and the posterior wall of the internal carotid artery is exposed on the anterolateral side. The cochlea and internal auditory canal are backward exposed. When dealing with tumors, especially tumors in the middle and posterior cranial fossa, it is necessary to avoid pulling the facial and auditory nerves, the cerebellum and the vein of Labbé. For tumors growing in petroclival region and across the middle and posterior cranial fossa, the visual field ranges superior to inferior from the posterior clinoid processes, the oculomotor nerve and the facial nerve.

The operation is mainly performed by six doctors with more than 10 years of experience in neuro-tumor surgery. The postoperative management team is also very experienced.

According to Simpson's classification of the degree of surgical resection of PM, PM cases can be divided into Simpson I-V. The degree of resection can be divided into gross-total resection (GTR), subtotal resection (STR) and partial resection. Simpson grade I or II resections were grouped as GTRs, whereas Simpson grade III, IV, and V resections were grouped as STRs (15).

All patients with significant residual tumors on MRI scans are recommended for radiosurgery or radiation therapy and are advised to consult a radiation oncologist.

### Statistical Analyses

Statistical analysis was performed with IBM SPSS Statistics 22 (IBM, Armonk, NY, USA), and the significance was set to *P* < 0.05. The quantitative data were analyzed by the Kolmogorov-Smirnov test to determine whether they were normal. Normal data are expressed as the mean and standard deviation (SD), and non-normal data were expressed as the median and interquartile range (IQR). Qualitative data are expressed as a percentage or ratio. The Mann-Whitney U test was used for the nonparametric test.

## Results

### Clinical Manifestations

Among all patients, 55 (53.9%) had headache or dizziness, 11 (10.8%) visited the hospital because of aphasia, and 35 (34.3%) were referred to the neurosurgery department because of a medical examination. One patient (1%) was treated for epilepsy. Through physical examination, 67 patients (65.7%) were found to have varying degrees of cranial nerve injury. The duration of symptoms ranged from 10 days to 156 months, and 30 patients had hypertension, diabetes or chronic respiratory disease. Of all PM cases, the UC type accounted for 29.4% (30 cases), the CS type accounted for 24.5% (25 cases), the TE type accounted for 30.4% (31 cases), and the PA type accounted for 15.7% (16 cases) (Table 1).

### Tumor Size

**Figure 2:**
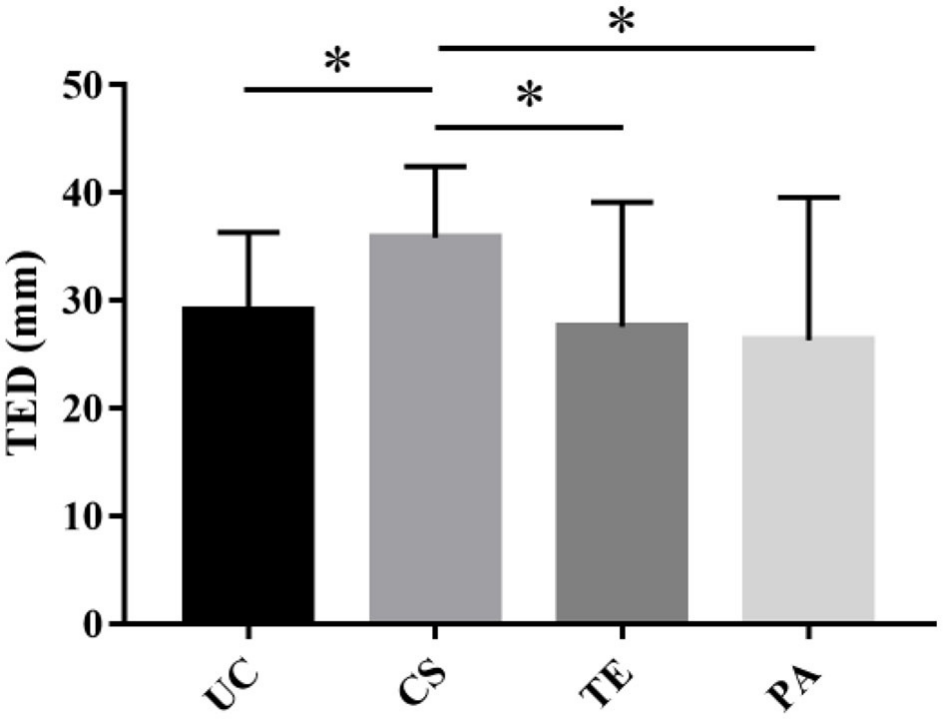
The tumor equivalent diameter (TED) of various PM subtypes. The TED of the CS type was significantly different from those of the other three types of PM (^*^P < 0.05).

The mean TED was 29.10 ± 10.3 mm. Most tumors were large-diameter tumors, and the average diameters of the UC, CS, TE, and PA types were 29.1 ± 6.47 mm, 35.8 ± 6.49 mm, 27.6 ± 11.32 mm, and 26.3 ± 12.8 mm, respectively. The TED of the CS type was significantly different from those of the other three types of PM (*P* < 0.05) (Table 1 and Figure 2).

### Selection of the Surgical Approach and Surgical Results

According to the preoperative imaging data, the location of the base of the tumor and the area of invasion, different surgical approaches were used.

#### UC Type

Thirty cases of UC-type PM were treated with three approaches: subtemporal, retromastoid, and the Kawase approach. The rates of Simpson grade I and II resection with the Kawase approach reached 23.33 and 23.33%, respectively, while the Simpson grade I resection rates with the subtemporal and retromastoid approaches reached 3.33% each, and the Simpson grade II resection rates reached 16.67 and 6.67%, respectively. The GTR rate of the Kawase approach was 46.66%, which was significantly different from that of the other two approaches (*P* = 0.044); however, the operation time was also relatively long, that is, 482.2 ± 66.19 min, whereas the operation times of the other two approaches were 468.5 ± 186.52 min and 323.2 ± 127.79 min, respectively. The total length of stay of the patients was 22.1 ± 3.45 d, 23 ± 13.13 d and 24.2 ± 9.52 d, respectively, with no significant differences (*P* > 0.05) (Tables 2, 3).

**Table 2:** Degree of resection of four tumor types in patients with PM.

**Types (surgical approach)**	**Degree of resection**				
	**Simpson I**	**Simpson II**	**Simpson III**	**Simpson IV**	**Simpson V**
**UC**					
Subtemporal	1	5	3	1	0
Retromastoid	1	2	2	0	0
Kawase	7	7	1	0	0
**CS**					
Subtemporal	0	0	11	4	0
Kawase	0	0	7	3	0
**TE**					
Retromastoid	1	4	2	1	0
Kawase	12	8	2	1	0
**PA**					
Retromastoid	0	5	1	1	0
Kawase	5	3	1	0	0

*CS, cavernous sinus type; CN, Cranial nerve; PA, petrous apex type; PM, Petroclival meningioma; TE, tentorium type; UC, upper clivus type*.

**Table 3:** Preoperative, postoperative and recent KPS of subtype.

**Tumor type**	**Approach**	**Overall (%)**	**Pre-KPS (median)**	**Operation time (min)**	**Post-KPS (median)**	**Recent KPS (median)**	**Hospital stay (d)**
UC	Subtemporal	10 (9.80)	68 ± 9.80 (65)	468.5 ± 186.51	74 ± 12.81 (70)	67.8 ± 22.3 (80)	22.1 ± 3.45
	Retromastoid	5 (4.90)	62 ± 22.27 (70)	323.2 ± 127.80	74 ± 27.28 (90)	68 ± 34.30 (80)	23 ± 13.13
	Kawase	15 (14.71)	68.6 ± 8.05 (70)	482.2 ± 66.2	75 ± 8.84 (70)	86 ± 24.44 (90)	24.2 ± 9.52
CS	Subtemporal	15 (14.71)	71.3 ± 10.87 (70)	350.87 ± 82.86	78 ± 10.46 (80)	86.8 ± 24.13 (90)	20 ± 3.25
	Kawase	10 (9.80)	69 ± 7 (70)	376.5 ± 105.18	77.5 ± 4.33 (80)	85 ± 15 (95)	24.8.8 ± 15.8
TE	Retromastoid	8 (7.84)	70 ± 10 (70)	414.5 ± 42.56	77.5 ± 6.14 (80)	85 ± 11.18 (90)	19.25.3 ± 3.86
	Kawase	23 (22.55)	62 ± 19.39 (70)	385.52 ± 81.24	74.8 ± 19.5 (80)	83.9 ± 27.30 (90)	24.2 ± 13.36
PA	Retromastoid	7 (6.86)	72.85 ± 6.99 (70)	412.43 ± 39.07	75.7 ± 9.03 (80)	88.57 ± 11.2 (90)	19.85 ± 0.83
	Kawase	9 (8.82)	70 ± 8.16 (70)	443.33 ± 73.71	83.3 ± 9.42 (80)	93.33 ± 9.42 (100)	16.44 ± 1.89
Total		102	68.72 ± 12.1 (70)	369.63 ± 116.96	76.3 ± 15.2 (80)	83.64 ± 24.78 (90)	22.58 ± 9.86

*CS, cavernous sinus type; KPS, Karnofsky performance scale; PA, petrous apex type; TE, tentorium type; UC, upper clivus type*.

#### CS Type

Twenty-five cases of CS-type PM were treated with the subtemporal and Kawase approach. There were no cases of Simpson grade I or II resection in either approach. Simpson III resection was achieved in 11 cases (73.33%) by the subtemporal approach, while grade III resection was achieved in seven cases (70%) by the Kawase approach. The chi-square test showed no significant difference between the two surgical approaches for CS tumor resection (*P* = 0.41). The time spent on the Kawase approach was 446.7 ± 75.86 min, while the time spent on the subtemporal approach was 350.87 ± 82.87 min; the difference was not significant (*P* = 0.24). The final hospital stays of the two approaches were 20.28 ± 3.25 d and 24.8 ± 15.8 d, respectively. The chi-square test showed no significant difference in length of hospital stay between the two approaches (*P* = 0.86) (Tables 2, 3).

#### TE Type

Thirty-one cases of TE-type PM were treated with the retrosigmoid and Kawase approaches. With the retrosigmoid approach, Simpson grade I resection was performed in 1 case (12.55%), and Simpson grade II resection was performed in 4 cases (50%). With the Kawase approach, Simpson grade I resection was performed in 12 cases (52.17%) and grade II resection was performed in 8 cases (34.78%). The tumor resection degree of the Kawase approach was higher than that of the retrosigmoid approach (*P* = 0.039). The operation time of the retrosigmoid approach was 414.5 ± 42.56 min, and the Kawase approach was 385.52 ± 81.24 min; there was no significant difference (*P* = 0.24). The hospitalization times of the two approaches were 19.25.6 ± 3.86 d and 24.3 ± 13.36 d, respectively. The chi-square test showed that there was no significant difference between the two groups (*P* = 0.23) (Tables 2, 3).

#### PA Type

The retrosigmoid and Kawase approach were used in 16 cases of PA-type PM. Simpson grade I resection was performed in 5 cases with the Kawase approach (31.25%), and grade II resection was performed in 3 cases (18.75%). In the case of the retrosigmoid approach, there were no Simpson I resections, but there were 5 cases (31.25%) of grade II resection, and 1 case (6.25%) each of grade III and IV resection. Comparatively, the grade I/II resection rate of the Kawase approach was higher, and it was more suitable for the resection of PA-type PM (*P* = 0.033). The operation times of the two approaches were 412.42 ± 39.07 min and 443.33 ± 73.71 min, respectively, with no significant difference (*P* = 0.457). However, the hospitalization time of patients with the Kawase approach was shorter than that of patients with the retrosigmoid approach (*P* = 0.002) (Tables 2, 3).

### Follow-Up and Statistical Analysis

Except for 5 patients who were lost to follow-up, the remaining 97 patient all visited the hospital for review 6 months after the operation. Our study used the improvement in the KPS score (postoperative KPS score-preoperative KPS score) as an indicator to compare the benefits in patients who underwent different surgical approaches for various tumor subtypes.

**Figure 3:**
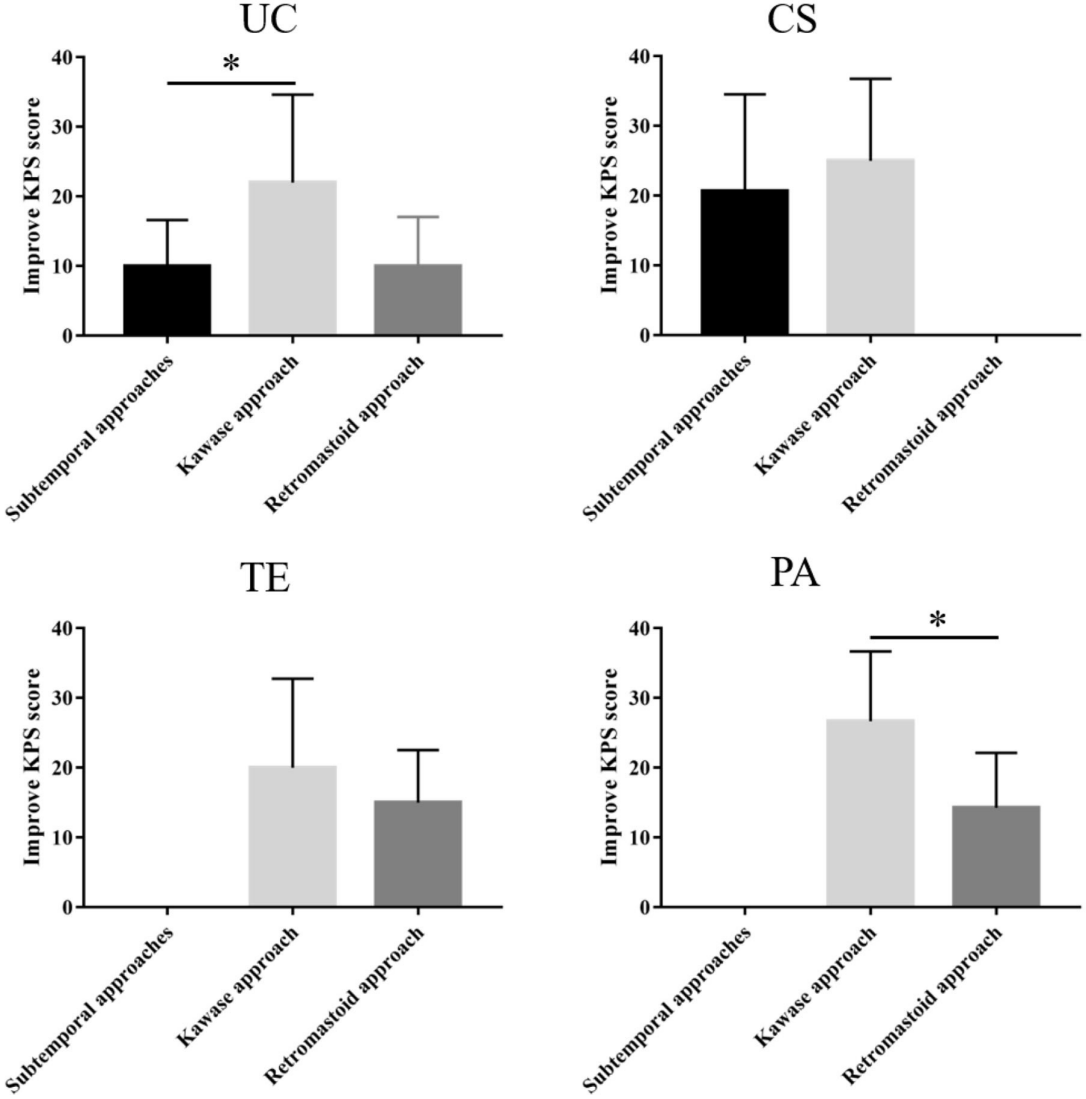
Different surgical approaches and KPS score improvement for each tumor subtype. The Kawase approach significantly improved the KPS scores at follow-up compared with the other two approaches for the UC and PA type (^*^P < 0.05).

Among the three approaches used for the **UC** type, the subtemporal approach yielded a KPS score that was 68 ± 9.8 (median 65) before the operation and 74 ± 12.81 (median 70) and 67.8 ± 22.3 (median 80) at discharge and follow-up, respectively. The KPS score of the retrosigmoid approach was 62 ± 22.27 (median 70) before the operation; postoperatively, the symptoms improved, and the KPS score was 74 ± 27.28 (median 90) but then decreased at follow-up relative to discharge. The KPS score of the patients who underwent the Kawase approach was 68.6 ± 8.05 (median 70) before the operation, and the KPS scores at discharge and follow-up were 75 ± 8.84 (median 70) and 86 ± 24.44 (median 90), respectively. Therefore, the Kawase approach significantly improved the KPS scores at follow-up compared with the subtemporal approach (*P* < 0.05). However, there were few patients in the retrosigmoid approach group, and no significant difference was observed (Table 3 and Figure 3).

The KPS scores of the subtemporal and Kawase approaches for the CS type were 71.3 ± 10.87 (median 70) and 69 ± 7 (median 70) before the operation, 78 ± 10.46 (median 80) and 77.5 ± 4.33 (median 80) at discharge, and 86.8 ± 24.13 (median 90) and 85 ± 15 (median 95) at follow-up, respectively. Both approaches improved the KPS score during follow-up, but there was no difference between the two approaches (*P* = 0.034). Therefore, the two surgical approaches can improve the postoperative quality of life of patients for CS PM (Table 3 and Figure 3).

For the TE type, the preoperative KPS scores of the retrosigmoid and Kawase approach were 70 ± 10 (median 70) and 62 ± 19.39 (median 70), respectively. Symptoms improved after the operation and during follow-up, and the KPS scores were 85 ± 11.18 (median 90) and 83.9 ± 27.30 (median 90), respectively, at follow-up. Both approaches significantly improved the KPS score at follow-up, with no significant difference between the two approaches (*P* = 0.242) (Table 3 and Figure 3).

The PA type was resected mainly through the retrosigmoid and Kawase approaches. The preoperative KPS scores were 72.85 ± 6.99 (median 70) and 70 ± 8.16 (median 70), respectively. The postoperative symptoms improved, and the KPS scores increased to 75.7 ± 9.03 (median 80) and 83.3 ± 9.42 (median 80), respectively. The KPS scores increased to 88.57 ± 11.2 (median 90) and 93.33 ± 9.42 (median 100) at follow-up, respectively. The Kawase approach significantly improved the KPS scores at follow-up compared with the retrosigmoid approach (*P* < 0.05) (Table 3 and Figure 3).

At the same time, we also followed up on cranial nerve injuries that is an important evaluation index for the patient's condition. Compared with the patient's preoperative data, postoperative cranial nerve injury includes deterioration, new onset, improvement and unchanged. Among the UC-types PM, none of the three surgical methods caused aggravation or new occurrence of cranial nerve injury, and most of them improved symptoms (Table 4A). In CS-type PM, cranial nerve injury is concentrated in CN III-VI. The conditions of the two patients deteriorated, and both were the sixth pair of cranial nerve injuries (Table 4B). In the TE-type PM, the patient's symptoms mostly involve CN III-X. After surgery, the patient's cranial nerve injury has almost improved. Patients with worsening conditions all appeared in the fifth pair of cranial nerves (Table 4C). In the PA-type PM, none of the patients experienced deterioration. Cranial nerve injuries are mostly concentrated in CN V-VIII (Table 4D).

**Table 4A:** Recovery of UC type cranial nerve injury.

**Neruopathy**	**UC**								
	**Subtemporal**			**Retrosigmoid**			**Kawase**		
	**Improve**	**D/NO**	**No change**	**Improve**	**D/NO**	**No change**	**Improve**	**D/NO**	**No change**
CN II	–	–	1	–	–	–	–	–	–
CN II	–	–	–	–	–	–	–	–	–
CN IV	–	–	–	2	–	–	–	–	–
CN V	1	–	–	2	–	–	–	–	–
CN VI	–	–	–	–	–	–	–	–	–
CN VII	–	–	–	–	–	–	2	–	–
CN VIII	–	–	–	–	–	–	2	–	–
CN IX	–	–	–	1	–	–	1	–	–
CN X	1	1	–	–	–	–	1	–	–
CN XI	–	–	–	–	–	–	1	–	–
Ataxia	–	–	–	–	–	–	1	–	1
Dizziness	3	–	–	3	–	–	2	–	2

*CN, Cranial nerve; D, Deterioration; NO, new onset; UC, upper clivus type*.

**Table 4B:** Recovery of CS type cranial nerve injury.

**Neruopathy**	**CS**					
	**Subtemporal**			**Kawase**		
	**Improve**	**D/NO**	**No change**	**Improve**	**D/NO**	**No change**
CN II	–	–	1	1	–	–
CN III	2	–	–	–	–	1
CN IV	1	–	1	1	–	–
CN V	2	–	–	1	–	–
CN VI	1	1	–	1	1	1
CN VII	–	–	–	1	–	–
CN VIII	–	–	–	–	–	–
CN IX	–	–	–	–	–	–
CN X	–	–	–	–	–	–
CN XI	–	–	–	–	–	–
Ataxia	–	–	–	–	–	–
Dizziness	3	–	–	5	–	2

*CN, Cranial nerve; D, Deterioration; NO, new onset; CS, cavernous sinus type*.

**Table 4C:** Recovery of TE type cranial nerve injury.

**Neruopathy**	**TE**					
	**Retrosigmoid**			**Kawase**		
	**Improve**	**D/NO**	**No change**	**Improve**	**D/NO**	**No change**
CN II	–	–	–	–	–	–
CN III	1	–	–	–	–	–
CN IV	1	–	1	–	–	–
CN V	1	1	–	3	1	2
CN VI	–	–	1	–	–	1
CN VII	–	–	–	1	–	–
CN VIII	1	–	–	3	–	–
CN IX	–	–	–	1	–	–
CN X	1	–	–	2	–	–
CN XI	–	–	–	–	–	–
Ataxia	–	–	–	–	–	–
Dizziness	3	–	–	4	–	2

*CN, Cranial nerve; D, Deterioration; NO, new onset; TE, tentorium type*.

**Table 4D:** Recovery of PA type cranial nerve injury.

**Neruopathy**	**PA**					
	**Retrosigmoid**			**Kawase**		
	**Improve**	**D/NO**	**No change**	**Improve**	**D/NO**	**No change**
CN II	–	–	–	–	–	–
CN III	–	–	–	–	–	–
CN IV	–	–	–	–	–	–
CN V	3	–	–	2	–	–
CN VI	1	–	–	–	–	1
CN VII	1	–	1	–	–	–
CN VIII	1	–	–	1	–	1
CN IX	–	–	–	1	–	–
CN X	–	–	–	–	–	–
CN XI	1	–	–	–	–	–
Ataxia	2	–	–	1	–	–
Dizziness	3	–	–	4	–	–

*CN, Cranial nerve; D, Deterioration; NO, new onset; PA, petrous apex type*.

## Discussion

The incidence of petroclival tumors is relatively low, accounting for 0.15% of all intracranial tumors, 10% of intracranial meningiomas, and 2% of posterior fossa (1–4). More females are affected than males; the ratio of males to females is ~1:2. In this series, there were 21 males and 81 females, and the proportion was 1:3.9. The average age of onset was 52 years old, and the peak of onset occurred at 48.5–56 years of age. Other cases described in the literature (2, 16, 17) also report a higher incidence rate in women. Van Havenbergh et al. found through retrospective analysis (12, 18, 19) that PM is benign, but the long-term prognosis is poor. However, many meningiomas may stop growing after menopause. This characteristic is particularly important for evaluating the degree of benefit to elderly women when choosing surgical treatment.

There are many types of surgical procedures and improved approaches to the petroclival region that have gone through different stages of development. Kawase (20) states that the best surgical approach for petroclival tumors should: a. be the shortest approach with sufficient exposure and a wide working angle; b. cause low retraction to the cerebrum; c. not cross the important veins; d. allow possible detachment of tumor feeders (tentorial artery, middle meningeal artery); e. not cross the important cranial nerves and auditory organs; and f. be an easily learned surgical technique.

Patients with UC-type PM are prone to vertigo, ataxia and other symptoms due to the brain stem and cerebellum being squeezed by the tumor. The purpose of the operation is to relieve the compression of the brainstem in a timely manner (21). For UC-type PM, because the tumor mainly originates between cranial nerves V-VI of the anterolateral cerebellopontine, attention must be paid to prevent the trigeminal nerve from being flattened and squeezed under the tentorial cerebellum when creating the tentorial incision. Incision of Meckel's cave is essential in the event of tumor invasion to mobilize the trigeminal nerve root and remove lesions medial to the nerve (13, 20). Excessive trigeminal nerve root traction caused by tumors can limit tumor invasion to the facial and auditory nerves to some extent and protect the arachnoid of the cerebellopontine angle cistern (22, 23). The Kawase approach can reduce the disturbance of the facial and acoustic nerves and the complications caused by injury to the acoustic nerves. At the same time, the Kawase approach can reduce the disturbance of the facial and auditory nerves and the complications caused by injury of the acoustic nerves by grinding the bone of the skull base. At the same time, the operation has a wide field of vision, can directly reach the prepontine cistern and upper clivus, does not need to pull the cerebellum, and can deal with the lesions of the middle and posterior cranial fossa at the same time. When the retrosigmoid approach is used to remove the tumor, it is necessary to separate the facial and auditory nerves, which increases the possibility of iatrogenic nerve injury. In our group, when using the Kawase approach for the UC type, the Simpson I/II GTR reached 46.66%. The KPS score increased from 68.6 ± 8.05 (median 70) preoperatively to 86.8 ± 24.44 (median 90) postoperatively. Compared with the other two approaches, the postoperative quality of life of the patients was significantly improved. Therefore, the Kawase approach is more suitable for the UC-type PM.

CS-type PM causes facial numbness, abducent nerve paralysis, ophthalmoplegia and other symptoms, of which abducens nerve palsy is the most common. This type of tumor may originate from Meckel's cave or the posterolateral CS, resulting in corresponding symptoms of nerve damage in the early stage of the disease (24, 25). Therefore, CS-type PM surgery is the most difficult because the tumor invades the CS, the dura mater is compressed backward by the tumor, the space for subdural surgery is small, and the cranial nerve and ICA are easily wrapped by the tumor (22). Radical resection is impossible, and only partial resection of the subdural tumor can be obtained. Improving quality of life and reducing the risk of extensive nerve and vascular injury caused by reoperation has become a must for surgeons. At present, craniotomy combined with postoperative radiotherapy is advocated by an increasing number of surgeons (26, 27). In our group, there were no cases of Simpson grade I and II resection for CS-type PM with the subtemporal approach or Kawase approach, and grade III resection reached a rate of more than 70%. Neither of the two approaches could completely solve the problem of tumor and nerve and vascular entrapment in the CS. This may be explained because the tumor volume of this subtype is larger than that of the other subtypes, and the tumor has certain invasiveness. Furthermore, the intracranial part of the operation is relatively complex, which could potentially increase the occurrence of complications.

The common symptoms of TE-type PM are facial numbness, hearing impairment and increased intracranial pressure, such as headache and dizziness. Both supratentorial and infratentorial growth of the tumor will show obvious symptoms of intracranial hypertension, such as headache and dizziness. However, the tumor usually involves the tentorial notch of the cerebellum, resulting in displacement of the trigeminal nerve and the superior cerebellar artery to the brainstem and downward displacement of the facial acoustic nerve. The main body of the TE PM is located in the posterior cranial fossa, but the tumor can grow supratentorially through the tentorial hiatus or invade the middle cranial fossa through Meckel's cave. According tumor growth, the opening can be partially or completely cut along the petrosal crest to the free margin. However, the trochlear nerve runs under the tentorium of the cerebellum, so to avoid damage to the trochlear nerve during incision, the free edge of the trochlear nerve should be pulled out laterally and upward to expose the position where the trochlear nerve enters (28). For this type of tumor, it is necessary to fully expose the main body of supratentorial and infratentorial tumors and to observe the blood supply of the brain stem and cerebellum. The Kawase approach can provide the extensive exposure needed for supratentorial, infratentorial and middle cranial fossa resection, reduce intraoperative bleeding and increase the probability of radical resection. However, for those PMs that do not invade the middle cranial fossa, the retrosigmoid approach is another alternative. Because of its full exposure of the posterior cranial fossa, simple approach, relatively low trauma, short operation time, early exposure of the tumor and base, relatively few complications and rapid postoperative recovery, the retrosigmoid approach is widely known and used (29, 30). In this type of PM, retrosigmoid approach Simpson I/II resection (GTR) was performed in 5 cases (62.55%), Kawase approach Simpson I/II resection (GTR) was performed in 12 cases (87.95%), and III/IV (STR) was performed in 3 cases (9.68%). Overall, the total resection rate of GTR by the Kawase approach was higher than that of the retrosigmoid approach. The operation time of the retrosigmoid approach was 414.5 ± 42.56 min, whereas the operation time of the Kawase approach was 385.52 ± 81.24 min. There was no significant difference between the two approaches (*P* = 0.24). Therefore, we believe that the Kawase approach is also suitable for the resection of TE-type PM.

PA-type PM mainly induce symptoms caused by trigeminal nerve compression. Because the PA-type PM is mainly located in the postero-interior area of the middle cranial fossa, near the semilunar ganglion of the trigeminal nerve, the early onset of the disease is often characterized by abnormal sensation and pain in the distribution area of the trigeminal nerve, and with disease progression, the motor function of the trigeminal nerve also decreases. When the tumor oppresses the CS, it may produce the same symptoms related to eye movement and abducent nerve paralysis as the CS-type PM. Tinnitus and hearing impairment similar to tumors in the CPA region may occur when the intrusive petrous bone oppresses the eustachian canal and the posterior cranial fossa. Typical PA-type PM pushes cranial nerve V laterally, a portion of which invades the internal auditory canal, while petrous apical meningioma pushes the nerve medially. Both cases may yield facial pain or sensory disturbance after trigeminal nerve injury; however, PA-type PM rarely invades Meckel's cave but directly oppresses the brain stem area of the trigeminal nerve and rarely extends to the middle cranial fossa, the parasellar region or the dura (13, 25). For this type of tumor, it is necessary to remove the tumor to the maximum extent while ensuring that the function of the facial and acoustic nerves is not impaired, or at least retain functional hearing. In the Kawase approach the petrous bone can be ground to the maximum without damaging the important structures, and the midline of the clival and the area above the facial acoustic nerve can be fully exposed (30). Therefore, it is appropriate to use the Kawase approach for PA-type PM with invasion of the internal auditory canal, but it is necessary to consider injury to the superficial petrosal nerve and temporal lobe vein. The retrosigmoid approach is not only unable to remove bone from the epidural but also needs to pass through many nerves and blood vessels, especially the facial and auditory nerves, although this approach can provide a wider field of vision of the posterior cranial fossa. However, it is necessary to keep a distance from the facial and auditory nerves. In this case, the total resection rate for Simpson grade I/II with the Kawase approach was 50% and that with the retrosigmoid approach was 31.25%. The resection rate of the grade I/II Kawase approach was higher (*P* = 0.033), and this approach is more suitable for PA-type PM.

## Conclusions

According to the tumor origin found on imaging examination combined with clinical symptoms, intraoperative findings, and Kawase's PM classification system, we divided PM into four unique types. The advantages of the four-way classification and the corresponding surgical approaches could provide maximal safe resection and improved KPS scores. The Kawase approach is more suitable for PM tumors, especially for UC- and PA-type PM.

Therefore, understanding the classification of PM and the anatomical relationship between various types of tumors and peripheral nerves and blood vessels can help formulate the most beneficial surgical methods for patients and improve their survival rate and quality of life.

## Limitations and Prospects

Because this was a single-center clinical sample analysis, the sample size of each subtype was not large. In addition, because the surgeon had fewer attempts for more access, this may have limited the access to the study sequence, which would affect the overall analytical results. However, we hope that our research can provide some clinical ideas for the surgical novice, and at the same time, we will use the corresponding surgical approach based on the predictability of tumor classification according to our own research results.

## Data Availability Statement

The raw data supporting the conclusions of this article will be made available by the authors, without undue reservation.

## Ethics Statement

The studies involving human participants were reviewed and approved by Ethics Committee for Human Experiments of Zhengzhou University. Written informed consent for participation was not required for this study in accordance with the national legislation and the institutional requirements. Written informed consent was obtained from the individual(s) for the publication of any potentially identifiable images or data included in this article.

## Author Contributions

YL and QG contributed to prepare the manuscript and the statistical analysis. FG put forward the concept of the study and designed the study. ZL reviewed the manuscript. HJ, NW, DX, FW, AG, and WZ contributed to the data acquisition, analysis, and interpretation. All authors read and approved the final manuscript.

## Conflict of Interest

The authors declare that the research was conducted in the absence of any commercial or financial relationships that could be construed as a potential conflict of interest.
